# Correlation between the timing of diagnostic ureteroscopy for upper tract urothelial cancer and intravesical recurrence after radical nephroureterectomy

**DOI:** 10.3389/fonc.2023.1122877

**Published:** 2023-03-22

**Authors:** Zhenkai Luo, Binbin Jiao, Caixia Su, Hang Zhao, Yangxuanyu Yan, Yijin Pan, Jian Ren, Guan Zhang, Zhenshan Ding

**Affiliations:** ^1^ Graduate School of Peking Union Medical College and Chinese Academy of Medical Sciences, Beijing, China; ^2^ Department of Colorectal Surgery, National Cancer Center/National Clinical Research Center for Cancer/Cancer Hospital, Chinese Academy of Medical Sciences and Peking Union Medical College, Beijing, China; ^3^ Department of Urology, Beijing Chao-Yang Hospital, Capital Medical University, Beijing, China; ^4^ School of Public Health, Peking University, Beijing, China; ^5^ Department of Urology, China-Japan Friendship Hospital, Beijing, China; ^6^ China-Japan Friendship School Clinical Medicine, Peking University, Beijing, China

**Keywords:** upper tract urothelial cancer, diagnosis ureteroscopy, intravesical recurrence, the timing of ureteroscopy, nephroureterectomy

## Abstract

**Objective:**

We aimed to evaluate the effect of the timing of diagnostic ureteroscopy (URS) on intravesical recurrence (IVR) following radical nephroureterectomy (RNU).

**Patients and methods:**

The clinical data of 220 patients with upper tract urothelial carcinoma (UTUC) treated with RNU at our center from June 2010 to December 2020 were retrospectively analyzed. According to the timing of the URS, all patients were divided into three groups: the no URS group, the 1-session group (diagnostic URS immediately followed by RNU), and the 2-session group (RNU after diagnostic URS). Additionally, we analyzed IVR-free survival (IVRFS) using the Kaplan-Meier and Cox proportional regression methods.

**Results:**

The median follow-up period of these 220 patents was 41 (range: 2-143) months. Among them, 58 patients developed IVR following RNU. Kaplan-Meier curve displayed a significantly higher IVR rate in both treatment groups than in the no-URS group (*p*=0.025). In the subgroup of patients with renal pelvis cancer, the incidence of IVR was significantly higher in both treatment groups than in the group without URS (*p*=0.006). In univariate Cox proportional regression analysis, the two treatment groups were risk factors for IVR compared to the no-URS group [*p*=0.027, hazard ratio (HR): 1.93, 95% confidence interval (CI): 1.08-3.46]. The two-stage group (*p*=0.032, HR: 1.98, 95% CI: 1.08-3.65), positive urine pathology (*p*<0.001, HR: 8.12, 95% CI: 3.63-18.15), adjuvant chemotherapy (*p*<0.001, HR: 0.20, 95% CI: 0.10-0.38), and positive margin (*p*<0.0001, HR: 7.50, 95% CI: 2.44-23.08) were all identified as independent predictors in the multivariate.

**Conclusion:**

This study revealed that delayed RNU following diagnostic URS may increase the risk of postoperative IVR in patients with UTUC, preoperatively positive uropathology, and positive surgical margin were risk factors for IVR after RNU, while early postoperative chemotherapy may effectively prevent IVR. Delay of RUN after URS could increase the risk of IVR.

## 1 Introduction

Upper tract urothelial carcinoma (UTUC) is a rare and aggressive malignant disease (1), accounting for approximately 5–10% of urothelial tumors, with an estimated incidence of 1-2 cases per 100,000 people annually in Western countries (2, 3). The gold standard for localized UTUC therapy currently is radical nephroureterectomy (RUN) with partial bladder cuff excision, which is a relatively large and challenging urological surgical technique (4). However, 22-47% of UTUC patients will experience intravesical recurrence (IVR) after RNU, which will lead to higher treatment costs for UTUC patients (5). Meanwhile, this will cause significant damage to the patient and lead to a reduced quality of life due to the need to remove the kidney, ureter and part of the bladder. Therefore, it is especially crucial to accurately diagnose UTUC patients before radical surgery. Recently, diagnostic ureteroscopy (URS) has emerged as a powerful diagnostic tool for UTUC due to the inability to accurately detect occult or tiny lesions in imaging examinations (2, 4). Therefore, according to the recommendations of the European Association of Urology (EUA), URS should be carried out in the preoperative assessment of any UTUC patient, and follow-up cystoscopy should be carried out to identify IVR in patients who receive radical nephroureterectomy (RUN) (6). Although some studies have suggested that URS surgery and related procedures may be a potential risk of tumor cells implanted into the bladder, leading to bladder recurrence after RNU surgery (2, 4, 7, 8), other investigations have demonstrated that there is no connection between URS and IVR (9). Thus, the effects of URS on IVR after RUN are still unclear. In addition, most previous studies have focused on the effect of ureteroscopy on IVR, and few studies have investigated whether the timing of ureteroscopy also affects the occurrence of IVR (10), and the correlation between the timing of URS before RNU and IVR has not been specifically evaluated. Loizzo et al. argued that diagnostic URS should not represent a ‘one-fits-all’ tool, but instead be offered following a risk-stratified approach (11). Based on the above reasons, we attempt to ascertain through retrospective analysis if diagnostic URS affects the incidence of IVR and whether the timing of URS and RNU also has an influence on IVR.

## 2 Methods

### 2.1 Study population

This study was authorized by the Institutional Review Board of China-Japan Friendship Hospital. Written informed consent for medical research was obtained from all patients before inclusion in this study. All experiment procedures, including data collection and management, were performed following relevant guidelines and regulations. All procedures involving human participants were complied with the Helsinki declaration and its later amendments or comparable ethical standards. Patients who received RNU at our center between January 2009 and January 2020 and had primary UTUC were the subjects of a prospective data collection. The inclusion criteria were determined as follows: (1) patients with pathologically confirmed UTUC; (2) patients with primary disease; (3) patients who underwent RNU combined with cystic sleeve resection and (4) patients who underwent URS or not. The exclusion criteria were: (1) patients with bilateral UTUC; (2) patients who received a cystectomy and no RNU; and (3) patients with metastatic carcinoma.

### 2.2 Data collection and follow-up regimen

Demographic, operative, and clinical data were recorded, including patient basic characteristics, preoperative examination [creatinine, neutrophil-to-lymphocyte ratio (NLR), lymphocyte-to-monocyte ratio (LMR), hydronephrosis, urine pathology, tumor characteristics, postoperative condition, overall survival (OS), IVR, extraurothelial recurrence (EUR), etc.]. Tumor multifocality was defined as the synchronous occurrence of two or more pathologically confirmed tumors in any upper urinary tract location. All surgically removed tumor specimens are typically staged using the International Union Against Cancer (UICC)/American Joint Committee on Cancer (AJCC) TNM classification (12) and graded according to the 2016 World Health Organization (WHO) classification (13). Patients were divided into 3 groups based on the timing of URS: the no URS group, the 1-session group and the 2-session group. The no URS group skipped the diagnostic URS; the 1-session group received a diagnostic URS followed by RNU immediately on the same day; the 2-session group had RNU at a median time of 8 days after URS.

Patients were usually followed up every 3-4 months in the first year after RNU, every 6 months from the second to the fifth year, and annually thereafter. Follow-up visits include history, physical examination, routine bloodwork, urinary cytology, chest radiography, cystoscopy and radiographic evaluation of the contralateral upper urinary tract. Imaging evaluations using computed tomography (CT) or magnetic resonance imaging (MRI) were performed every six months for the first five years and annually thereafter. Chest computed tomography or magnetic resonance imaging was performed when clinically indicated. If cystoscopy revealed suspicious lesions, cystoscopic biopsy and subsequent transurethral cystectomy were performed. IVR was defined as pathologically diagnosed uroepithelial carcinoma of the bladder after cystoscopic biopsy or transurethral resection of the bladder tumor.

### 2.3 Statistical analysis

Qualitative data were expressed as frequency tables, and non-normally distributed quantitative data were expressed as median values with ranges. The chi-square test for categorical variables and the variance test for continuous variables were used to compare the clinicopathological characteristics of the three groups of patients. The Kaplan-Meier curve and log-rank test were performed to evaluate and compare the effect of URS on IVR, OS and EUR in all patients. Univariate and multivariate Cox proportional hazard analyses were used to assess the effects of URS and other factors on IVR. Following univariate analysis, factors with *p*<0.2 were included in multivariate models and then reverse elimination was performed to identify the factors with the highest correlation to IVR-free survival (IVRFS) and OS. All *p* values were obtained from two-sided tests, a *p* value less than 0.05 was considered a statistically significant difference. R software (Version 4.1.2) and IBM SPSS Statistics (Version 24) were utilized to complete all statistical analyses and figures.

## 3 Results

### 3.1 Baseline characteristics

We finally included 220 patients with UTUC, 86 of whom did not receive URS, 22 underwent both URS and RNU, and the remaining 112 underwent URS and RNU performed separately. Biopsies were taken from these 134 patients who underwent URS. The median follow-up period of these 220 patents was 41 (range: 2-143) months. There were no statistical differences in the basic characteristics of the three groups, except for a significant difference in tumor location between the three groups (P<0.01) (**Table 1**). When underwent RUN, 58 patients (26.4%) developed intravesical recurrence, including 19.8% (17/86) in the no-URS group, 22.7% (5/22) in the 1- session group, and 32.1% (36/112) in the 2-session group. However, the difference in IVR rate among the 3 groups was not statistically significant (*p*=0.14).

**Table 1:** Association between clinicopathologic features and preoperative URS in UTUC.

Characteristic	No URS	1-Session	2-Session	*p* value
	N=86	N=22	N=112	
**Age (years)**	67 (38-86)	68 (45-79)	68 (40-83)	0.82
Sex				0.16
Male	36 (41.9%)	14 (63.6%)	48 (42.9%)	
Female	50 (58.1%)	8 (36.4%)	64 (57.1%)	
**BMI**	24.70 (16.42-40.00)	24.19 (19.84-33.13)	24.32 (16.44-33.25)	0.70
History of hypertension				0.76
No	46 (53.5%)	11 (50.0%)	54 (48.2%)	
Yes	40 (46.5%)	11 (50.0%)	58 (51.8%)	
History of diabetes				0.24
No	72 (83.7%)	15 (68.2%)	87 (77.7%)	
Yes	14 (16.3%)	7 (31.8%)	25 (22.3%)	
Laterality				0.67
Left	50 (58.1%)	12 (54.5%)	58 (51.8%)	
Right	36 (41.9%)	10 (45.5%)	54 (48.2%)	
Location				<0.01
Renal pelvis	51 (59.3%)	8 (36.4%)	38 (33.9%)	
Ureter	27 (31.4%)	14 (63.6%)	70 (62.5%)	
Both	8 (9.3%)	0 (0%)	4 (3.6%)	
Multifocality				0.97
Single	68 (79.1%)	17 (77.3%)	89 (79.5%)	
Multiple	18 (20.9%)	5 (22.7%)	23 (20.5%)	
Pathologic stage				0.85
pT1	48 (55.8%)	12 (54.5%)	56 (50.0%)	
pT2	17 (19.8%)	7 (31.8%)	28 (25.0%)	
pT3	17 (19.8%)	3 (13.6%)	24 (21.4%)	
pT4	4 (4.7%)	0 (0.0%)	4 (3.6%)	
Lymph node status				0.30
pN0/pNx	81 (94.2%)	22 (100.0%)	110 (98.2%)	
pN+	5 (5.8%)	0 (0.0%)	2 (1.8%)	
Tumor grade				0.34
Low	7 (8.1%)	4 (18.2%)	10 (8.9%)	
High	79 (91.9%)	18 (81.8%)	102 (91.1%)	
**Tumor size (cm)**	3.0 (0.4-8.5)	2.55 (1-7.5)	2.6 (0.2-9.5)	0.06
Margin status				0.63
Negative	84 (97.7%)	22 (100.0%)	107 (95.5%)	
Positive	2 (2.3%)	0 (0.0%)	5 (4.5%)	
Urine pathology				0.53
Negative	40 (46.5%)	10 (45.5%)	45 (40.2%)	
Tumor cell	13 (15.1%)	1 (4.5%)	20 (17.9%)	
Atypical	33 (38.4%)	11 (50.0%)	47 (42.0%)	
Hydronephrosis				0.31
≤2cm	59 (68.6%)	14 (63.6%)	65 (58.0%)	
>2cm	27 (31.4%)	8 (36.4%)	47 (42.0%)	
Adjuvant chemotherapy				0.37
No	47 (54.7%)	13 (59.1%)	52 (46.4%)	
Yes	39 (45.3%)	9 (40.9%)	60 (53.6%)	
Creatinine (μmol/L)				0.11
≤106	59 (68.6%)	19 (86.4%)	71 (63.4%)	
>106	27 (31.4%)	3 (13.6%)	41 (36.6%)	
**NLR**	4.13 (1.89-11.56)	4.43 (2.00-13.74)	4.03 (1.00-10.74)	0.42
**LMR**	1.64 (0.32-3.42)	1.53 (0.94-3.00)	1.59 (0.33-3.00)	0.76
**Survival time (months)**	43 (2-143)	49.5 (20-116)	38 (2-136)	0.07

UTUC, upper urinary tract cancer; URS, ureteroscopy; BMI, body mass index; NLR, neutrophil-to-lymphocyte ratio; LMR, lymphocyte-to-monocyte ratio

### 3.2 Analysis for predicting IVR following RNU

Patients were also stratified according to IVR (no IVR group and IVR group). Significant differences were observed between IVR and no-IVR patients in urine pathology (*p*<0.01), hydronephrosis (*p*=0.02), multifocality (*p*=0.01), adjuvant chemotherapy (*p*<0.01), margin status (*p*=0.02) (**Table 2**).

**Table 2:** Association between clinicopathologic features and IVR in UTUC.

Characteristic	With IVR	Without IVR	*p* value
	N=58	N=162	
**Age (years)**	67.5 (40-84)	67.5 (38-86)	0.84
Sex			0.51
Male	28 (48.3%)	70 (43.2%)	
Female	30 (51.7%)	92 (56.8%)	
**BMI**	24.31 (16.42-31.55)	24.61 (16.44-40.00)	0.37
History of hypertension			0.19
No	25 (43.1%)	86 (53.1%)	
Yes	33 (56.9%)	76 (46.9%)	
History of diabetes			0.42
No	48 (82.8%)	126 (77.8%)	
Yes	10 (17.2%)	36 (22.2%)	
Laterality			0.15
Left	27 (46.6%)	93 (57.4%)	
Right	31 (53.4%)	69 (42.6%)	
Location			0.72
Renal pelvis	24 (41.4%)	73 (45.1%)	
Ureter	30 (51.7%)	81 (50.0%)	
Both	4(6.9%)	8(5.0%)	
Multifocality			0.01
Single	39 (67.2%)	135 (83.3%)	
Multiple	19 (32.8%)	27 (16.7%)	
Pathologic stage			0.08
pT1	25 (43.1%)	91 (56.2%)	
pT2	19 (32.8%)	33 (20.4%)	
pT3	10 (17.2%)	34 (21.0%)	
pT4	4 (6.9%)	4 (2.5%)	
Lymph node status			0.68
pN0/pNx	57 (98.3%)	156 (96.3%)	
pN+	1 (1.7%)	6 (3.7%)	
Tumor grade			0.42
Low	4 (6.9%)	17 (10.5%)	
High	54 (93.1%)	145 (89.5%)	
**Tumor size (cm)**	3.0 (0.4-9.5)	2.5 (0.2-8.5)	0.24
Margin status			0.02
Negative	53 (91.4%)	160 (98.8%)	
Positive	5 (8.6%)	2 (1.2%)	
Urine pathology			<0.01
Negative	11 (19.0%)	84 (51.9%)	
Tumor cell	21 (36.2%)	13 (8.0%)	
Atypical	26 (44.8%)	65 (40.1%)	
Hydronephrosis			0.02
≤2cm	29 (50.0%)	109 (67.3%)	
>2cm	29 (50.0%)	53 (32.7%)	
Adjuvant chemotherapy			<0.01
No	45 (77.6%)	67 (41.4%)	
Yes	13 (22.4%)	95 (58.6%)	
Creatinine (μmol/L)			0.22
≤106	43 (74.1%)	106 (65.4%)	
>106	15 (25.9%)	56 (34.6%)	
**NLR**	4.14 (1.00-13.74)	4.03 (1.61-10.14)	0.39
**LMR**	1.52 (0.32-3.00)	1.6 (0.48-3.42)	0.35
URS			0.14
No URS	17 (29.3%)	69 (42.6%)	
1-Session	36 (62.1%)	76 (46.9%)	
2-Session	5 (8.6%)	17 (10.5%)	
**Survival time (months)**	39 (4-143)	42 (2-143)	0.76

UTUC, upper urinary tract cancer; IVR: intravesical recurrence; URS, ureteroscopy; BMI, body mass index; NLR, neutrophil-to-lymphocyte ratio; LMR, lymphocyte-to-monocyte ratio

In univariate cox proportional regression analysis, there was no significant difference in the time to diagnosis of URS and IVRFS (*p*=0.069). In multivariate cox regression analysis, urine pathology, hydronephrosis, tumor size, tumor multifocality, tumor pathological stage, tumor side, margin status, adjuvant chemotherapy, NLR, and LMR were all linked to IVRFS in univariate analysis, while only the 2-session (*p*=0.032), urine pathology positive (*p*<0.001), no adjuvant chemotherapy (*p*<0.001), margin positive (*p*<0.001) were independent predictors at the level of *P* value less than 0.05 in multivariate analysis (**Table 3**).

**Table 3:** Risk factors for predicting IVR following RUN for UTUC in 220 patients.

Characteristic	Univariate analysis		Multivariate analysis	
	HR (95% CI)	*p* value	HR (95% CI)	*p* value
Laterality				
Left	Reference		Reference	
Right	0.70(0.42,1.17)	0.168	0.93(0.53,1.64)	0.809
Multifocality				
Single	Reference		Reference	
Multiple	2.06(1.19,3.57)	0.010	1.15(0.61,2.16)	0.671
Pathologic stage				
pT1	Reference		Reference	
pT2	1.88(1.03,3.42)	0.039	2.16(1.14,4010)	0.018
pT3	1.32(0.63,2.75)	0.461	0.77(0.34,1.74)	0.531
pT4	2.61(0.91,7.52)	0.075	1.01(0.32,3.23)	0.981
**Tumor size**	1.11(0.95,1.29)	0.178	1.16(0.99,1.37)	0.073
Margin Status				
Negative	Reference		Reference	
Positive	4.94(1.96,12.43)	0.001	7.50(2.44,23.08)	<0.001
Urine pathology				
Negative	Reference		Reference	
Atypical	2.98(1.47,6.05)	0.004	2.79(1.32,5.88)	0.007
Tumor Cell	7.06(3.40,14.70)	<0.001	8.12(3.63,18.15)	<0.001
Hydronephrosis				
≤2cm	Reference		Reference	
>2cm	1.93(1.15,3.23)	0.013	1.45(0.84,2.51)	0.18
Adjuvant Chemotherapy				
No	Reference		Reference	
Yes	0.26(0.14,0.48)	<0.001	0.20(0.10,0.38)	<0.001
**NLR**	1.08(0.99,1.18)	0.054	0.98(0.88,1.10)	0.742
**LMR**	0.89(0.75,1.05)	0.167	0.94(0.77,1.14)	0.511
URS				
No URS	Reference		Reference	
1-Session	1.15(0.42,3.14)	0.782	0.72(0.24,2.19)	0.562
2-Session	1.93(1.08,3.46)	0.027	1.98(1.08,3.65)	0.028

UTUC, upper urinary tract cancer; IVR, intravesical recurrence; RUN, radical nephroureterectomy; URS, ureteroscopy; NLR, neutrophil-to-lymphocyte ratio; LMR, lymphocyte-to -monocyte ratio; HR, hazard ratio; CI, confidence interval.

### 3.3 Effect of the timing between URS and RNU on prognosis

**Figure 1:**
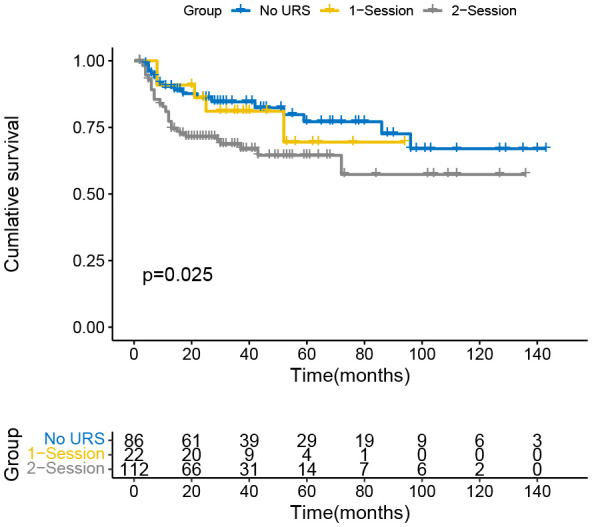
Survival analysis of intravesical recurrence in all patients.

**Figure 2:**
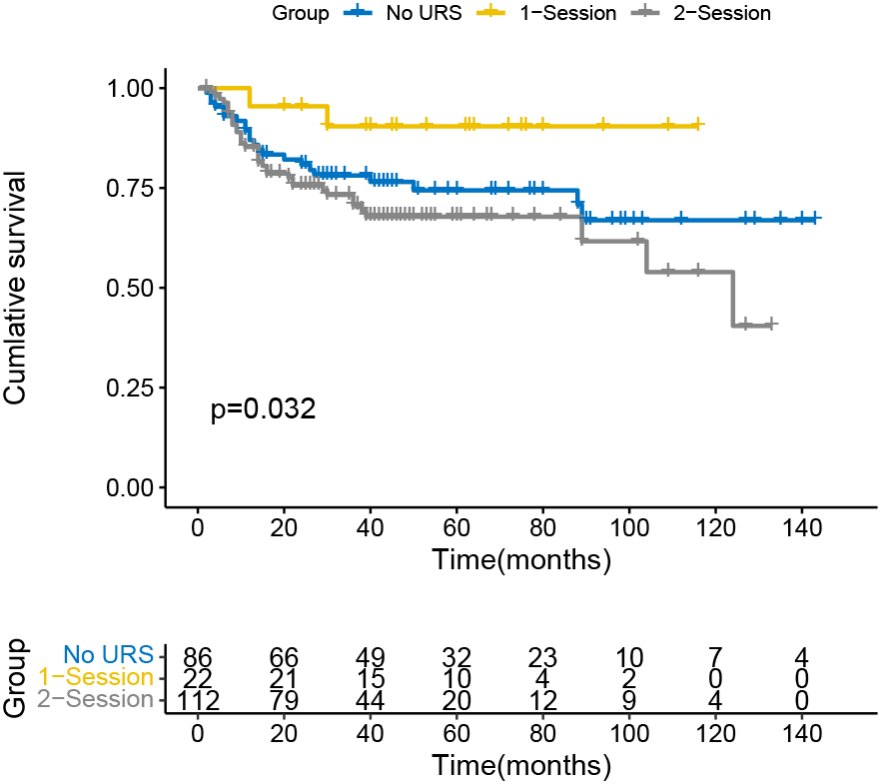
Survival analysis of extraurothelial recurrence in all patients.

**Figure 3:**
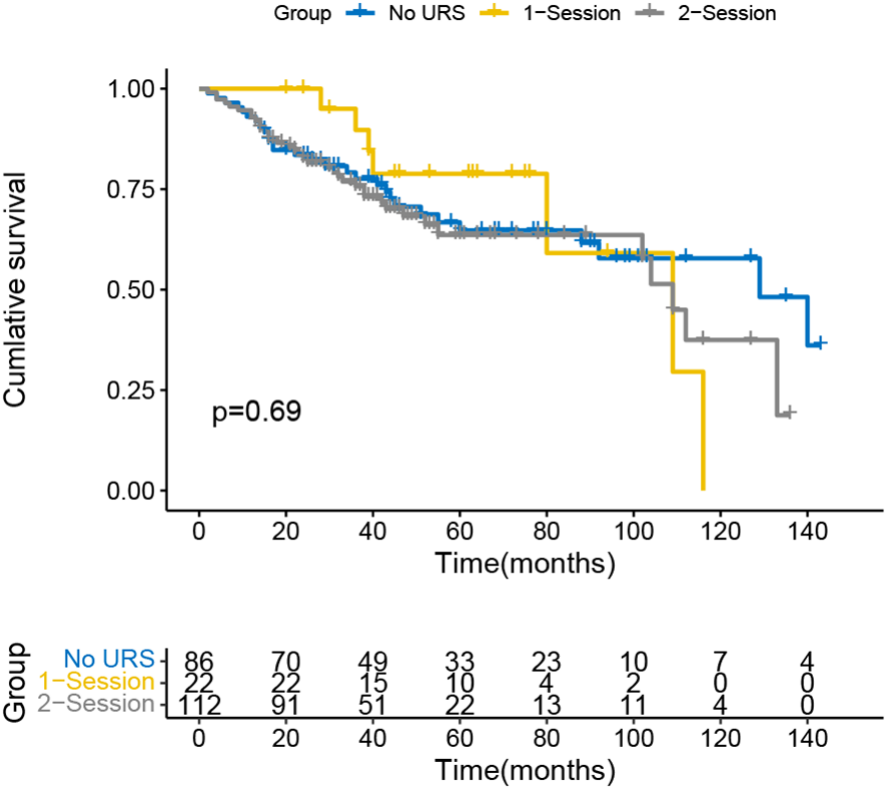
Survival analysis of overall survival in all patients.

**Figure 4:**
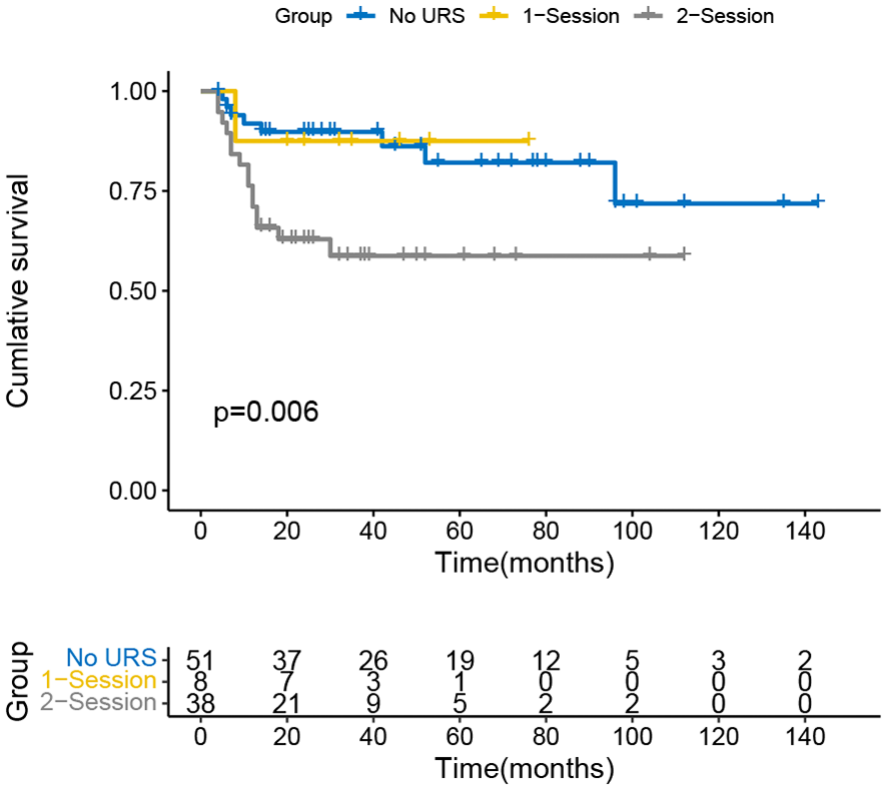
Survival analysis of intravesical recurrence in patients with renal pelvic carcinoma.

**Figure 5:**
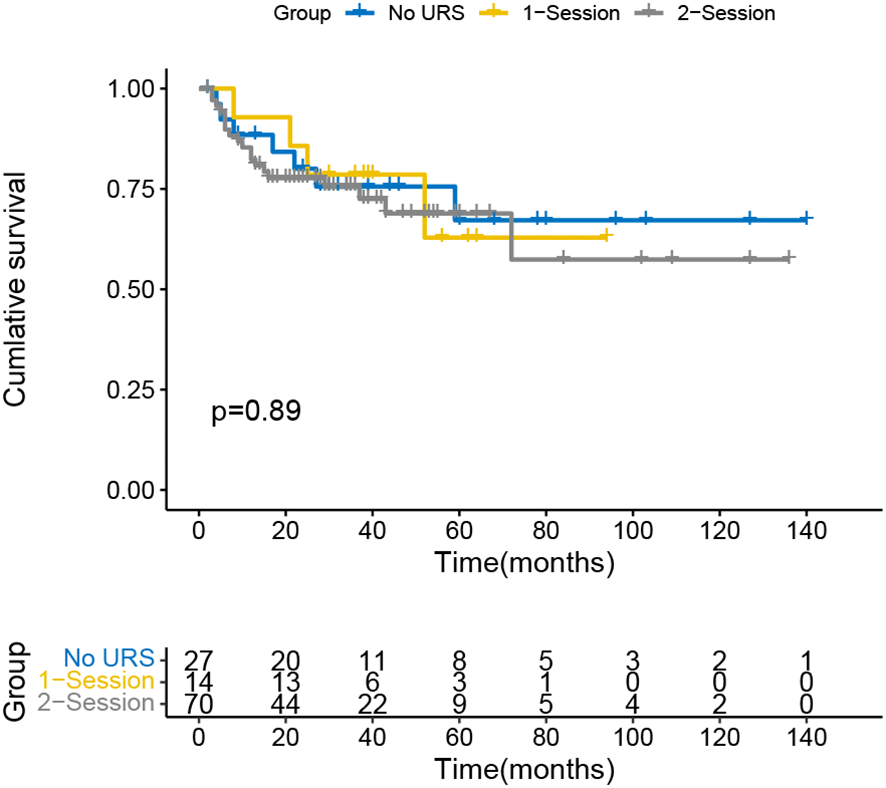
Survival analysis of intravesical recurrence in patients with ureteral carcinoma.

The Kaplan-Meier curve indicated that the IVR rate was considerably higher in the 2-session group than in the no URS group (**Figure 1**), but there was no significant difference between 1-session and others. In addition, there was also a statistically significant difference in the EUR rate between the 1-session and 2-session groups (**Figure 2**). No statistically significant difference for overall survival was recorded among the three groups (**Figure 3**). In the subgroup of patients with renal pelvic cancer, the IVR rate was significantly higher in the 2-session group than in the no URS group (**Figure 4**), but in the subset of patients with ureteral cancer, there was no statistically significant difference in IVR rate among the three groups (**Figure 5**).

## 4 Discussion

The mucosa of the renal pelvis, ureter, and bladder in the human urinary system are all composed of urothelial cells. UTUC is a urothelial carcinoma derived from the renal pelvis and ureter. RNU is the standard procedure for patients with high-risk UTUC, but the risk of postoperative IVR is relatively high. According to the findings of this study, the postoperative IVR following UTUC was 26.4% (58/220), which was comparable to the results of previous studies (14, 15). In addition, the results of this study suggested that RNU after URS, abnormal or malignant cells in the urine, positive surgical margins and the absence of postoperative adjuvant chemotherapy were independent risk factors for IVR after RNU in patients with UTUC.

Although RNU combined with sleeve resection of the bladder is the best strategy to treat localized UTUC, it is highly detrimental to the patients (6). A multidisciplinary systematic review of Wu et al. suggested the current level of evidence supporting neoadjuvant systemic therapy (NAST) for UTUC is relatively low (16). Therefore most patients could not avoid the complication of RUN, some patients even face problems such as decreased kidney function or dialysis treatment after surgery (17). As a result, early and prompt identification of UTUC is crucial. While urine flow cytology and imaging are frequent clinical techniques, histopathology is the gold standard for UTUC diagnosis. The ureteroscopic biopsy is a common preoperative histopathological diagnosis in clinical practice, which helps in preoperative risk grading of UTUC and provides evidence for clinical treatment decisions (18). Studies have demonstrated the sensitivity and accuracy of ureteroscopic biopsy for detecting malignant lesions in the upper urinary tract to be 82%-100% and 83%-100%, respectively (19). Nison et al. concluded that the data obtained during preoperative ureteroscopy (especially biopsy data) can help identify patients who may benefit from neoadjuvant chemotherapy or more extensive lymphadenectomy (3). However, preoperative diagnostic URS may be associated with postoperative IVR, some studies had pointed out that diagnostic URS increased the potential risk of tumor cell shedding leading to IVR during manipulation and flushing (2, 4, 5, 20), but others have shown that IVR is not affected by the diagnosis of URS (1, 3, 9). In the subgroup study involving individuals with or without a history of bladder cancer, Lee et al. did not discover that URS would result in an elevated risk of IVR (21). They hypothesized that manipulation-induced tumor detachment may not be the only cause of IVR, although the precise process is yet unclear.

Notably, patients with delayed RNU after diagnosis of URS may be at long-term risk of intracavitary tumor implantation (4). However, the majority of recent studies have concentrated on the issue of IVR following the URS combined RNU (1–5, 20, 21). According to our results, there was no statistically significant difference in IVR between the no URS group and the 1-session group, but the 2-session group was 1.98 times higher than the no URS group [HR=1.982(1.075-3.654), *p*=0.032]. The impact of intracavitary implantation on IVR is even more significant as diagnostic URS destroys tumor tissue and poses the risk of intracavitary implantation (22). During URS operation, pressure irrigation is usually carried out to obtain a clear examination and surgical view, and this step even leads to an increase in pressure even in the renal pelvis above 40cm H_2_O, disrupting the mucosal epithelial biological barrier (23). This causes the shedding of tumor cells and follows the direction of urine flow into the bladder, which can lead to recurrence. According to our findings, having tumor cells in the urine before surgery enhanced the IVR rate following surgery, which is consistent with the intraluminal seeding hypothesis. In addition, tumor cells can also be implanted in the bladder with the help of the ureteroscope. Investigators have identified the same p53 gene mutation in urothelial carcinomas of the upper and lower urinary tracts, that is, tumor cells containing the mutated gene were implanted into the bladder epithelium and migrated and spread in a monoclonal manner (24). It is important to note that uroepithelial carcinoma is also biologically more prone to implantation and spread than other types of tumors.

A study showed that the seeding and implantation of bladder cancer cells occurred during surgery, rather than before, because the migrating epithelium of the bladder was damaged during surgery and postoperative catheter retention (25). This undoubtedly provides favorable conditions for the implantation and spread of tumor cells, as they are more likely to adhere to the damaged epithelium, which is partly confirmed by the finding that IVR is more prevalent in the bladder neck (10). In our study, there was a statistically significant difference in IVRFS between the 2-session group and the no URS group in 97 patients with renal pelvis cancer (*p*=0.006), but not in 111 patients with ureteral cancer. Studies have demonstrated that patients with ureteral tumors have a worse prognosis than patients with pelvic tumors at the same stage and grade, which may be related to factors such as a closer distance between ureteral cancer and the bladder, and the faster flow of urine in the ureter than in the renal pelvis (8, 26). Furthermore, distal ureteral lesions had a positive correlation with IVR.

In terms of OS, there was no statistically significant difference among the three groups in this study (the no URS group, 1-session group and 2-session group), which was consistent with previous findings (27, 28). There was a statistically significant difference in EUR-free survival (EURFS) between the 1-session group and the 2-session group (*p*=0.032). We hypothesize that URS may promote metastasis by causing damage to the mucosal epithelium of the renal pelvis and ureter, which facilitates tumor cell invasion of the lymphatic and blood arteries.

Additionally, our study discovered that patients with UTUC may be more susceptible to developing IVR following RNU if they have positive urine cytology, a positive surgical margin, or no adjuvant chemoradiotherapy after surgery. Urine cytology can be used for cancer screening or follow-up. Positive urine cytology can assist in the early detection of urinary tract cancers because upper urinary tract tumor cells shed and enter the urine (29). According to the intraluminal dissemination hypothesis, positive preoperative urine cytology is a reasonable risk factor for IVR. Sakano et al. concluded that positive preoperative urine cytology predicted a lower degree of tumor differentiation and a poorer prognosis in patients with UTUC (30). In conclusion, positive urine cytology was an independent predictor of IVR, and in our investigation, the presence of abnormal and malignant cells in preoperative urine was also an independent predictor of IVR.

The study of Roupret et al. suggested that the positive surgical margins were associated with locally advanced pT stage, which is thought to be due to infiltration of surrounding soft tissues (mainly in the lateral wall, posterior wall and soft tissues around the prostate) in UTUC (6). In addition, the ureter has anatomical features such as the relative lack of retroperitoneal fat around it, therefore most of the patients (71.4%) with positive margins were ureteral carcinoma, and the end of the ureter is located in the trigone of the bladder (31). If the ureteral cancer is not eliminated during the operation, it will easily cause local recurrence. As a result, patients with positive surgical margins should be constantly monitored and IVR prevention measures should be implemented as soon as feasible. Besides, our study indicated that positive surgical margins were an independent risk factor for IVR. However, the intraoperative process (surgical method, surgical facility, operator’s experience), tumor biological characteristics (size/volume, invasiveness), and specimen processing effect, among other factors, determine whether the surgical margin is positive or not (32). Combining the influence of the small sample size, our results remain to be confirmed by further studies.

A study by Ito et al. revealed that immediate postoperative intravesical infusion of pirarubicin or (2″R)-4′-O-tetrahydropyranyl-doxorubicin (THP) significantly reduces IVR in patients with preoperatively positive urine cytology (25). These medications have a high rate of entry into the bladder tissue and can inhibit the replication and transcription of tumor cells, preventing the growth of new tumor cells (33). Postoperative prophylactic intravesical infusion chemotherapy is effective in reducing the IVR after RNU in UTUC patients and prolonging the time to first recurrence (34, 35). The results of our study support this conclusion.

Numerous investigations have revealed that numerous tumors were the most accurate predictor of IVR and an independent risk factor for the condition (36–38). Patients with multiple tumors tend to have poorer prognosis because this group of patients has more aggressive tumors or more severe diseases due to delayed diagnosis or treatment (39). However, there was no significant association between multiple tumors and IVR in this study, which may be related to the fact that most of the patients in this study were at an early stage.

We hope our finding will make contribution to this field and help clinicians decide on procedures for their patients. Although this study is rather novel, it still has the following limitations. The single-center retrospective study has a certain degree of selection bias. While evaluating particular aspects, a limited sample size on the basis of our strict inclusion criteria was used. The impact of intraoperative manipulation (surgical technique, surgical facility, operator experience) on postoperative results was not completely excluded in this research. Finally, regarding the data from a single center, these results should be discussed with cautious.

## 5 Conclusion

The results of this study revealed that delayed RNU following diagnostic URS may increase the risk of postoperative IVR in patients with UTUC, preoperatively positive uropathology, and positive surgical margin were risk factors for IVR after RNU, while early postoperative chemotherapy may effectively prevent IVR. Delay of RNU after URS could bring a higher risk of IVR in patients with UTUC. This study is helpful to assess the risk of postoperative IVR following RNU in URUC patients and for developing more individualized therapy and follow-up strategies, but more prospective, large-sample, multi-center studies are still required to confirm the findings.

## Data availability statement

The original contributions presented in the study are included in the article/supplementary material. Further inquiries can be directed to the corresponding authors.

## Ethics statement

The studies involving human participants were reviewed and approved by China-Japan Friendship hospital. The patients/participants provided their written informed consent to participate in this study.

## Author contributions

BJ and CS conceived of the presented idea. ZL made important contribution in the revision. ZL and BJ developed the theory and performed the computations. CS, YY, and YP verified the underlying data. BJ, CS, HZ, ZD, YY, YP, and JR collected the data, ZD and GZ supervises this study. All authors discussed the results and contributed to the final manuscript. All authors contributed to the article and approved the submitted version.
